# Effect of Music Tempo on Long-Distance Driving: Which Tempo Is the Most Effective at Reducing Fatigue?

**DOI:** 10.1177/2041669519861982

**Published:** 2019-07-15

**Authors:** Rui Li, Yingjie V. Chen, Linghao Zhang

**Affiliations:** School of Design, Jiangnan University, Wuxi, China; Department of Computer Graphics Technology, Purdue University, West Lafayette, IN, USA; School of Design, Jiangnan University, Wuxi, China

**Keywords:** attention, driving performance, fatigue, music tempo

## Abstract

This study investigated how music tempo impacted drivers’ fatigue and quality of attention in a long-distance monotonous highway environment. Sixteen drivers were enrolled in four sessions of real-road driving tests under the following four music conditions: no music, slow tempo, medium tempo and fast tempo. Specifically, the drivers’ electroencephalogram parameters and eye movement parameters were recorded to measure their extent of fatigue and quality of attention, respectively. Of the three tempos, medium-tempo music is the best choice to reduce fatigue and maintain attention for a long-distance driving. Slow-tempo music can temporarily boost the quality of attention, but after a long period of driving, it significantly deteriorates the driver’s levels of fatigue and attention. Fast-tempo music helps relieve driver fatigue but significantly deteriorates drivers’ attention after an extended driving time. This study offered practical references for drivers regarding the use of music to avoid fatigue, maintain attention and improve their driving safety. Based on previous theories of music and driving, we have explored the underlying mechanism of how music tempo maintains the alertness of drivers.

## Introduction

Vehicles have become one of the most natural acoustic environments for listening to music. Radios, CDs, smartphones and other mobile devices, such as the iPod, provide very convenient ways of listening to music while driving (Bedinger, 2016). In a survey of 1,780 British adults (Dibben & Williamson, 2007), approximately 68% of them reported listening to music or radios when driving, nearly half of them reported singing along with the music, 62% of them reported that music makes them feel relaxed and more comfortable and 25% of them reported that music helps them to be vigilant behind the wheel. Long-term driving on a monotonous road often induces boredom, fatigue, drowsiness and inattention. Experimental research and vehicle accident data show that both compromised vigilance fluctuation and overall vigilance significantly hinder driver safety under monotonous conditions (Folkard, 1997; Thiffault & Bergeron, 2003a). Listening to music has become the most common accompaniment for drivers to reduce boredom and maintain attention. According to the theory of compensation control, listening to music counteracts drivers’ fatigue, which thereby improves the effects of self-control strategies in driving safety (Ünal, Platteel, Steg, & Epstude, 2013). However, despite its wide acceptance, listening to music is not always safe for drivers (Patel, Ball, & Jones, 2008). The properties of different music elements may have different effects on the driver but have not been fully investigated.

Brodsky and Kizner (2012) argue that driver cognitive efficiency is influenced by a wide range of music elements, such as tone frequencies, instrumental ranges, arrangements, voicing textures, rhythm, intensity and tempos. In this project, we intended to study the effect of tempo. Obviously, the characteristics of a piece of music are determined by the interactions of many factors. Modern musicians have discovered and composed many new forms of music with much more complex interactions. To study all the possible interactions (combinations of variation on all the variables) is simply not feasible. However, music theories have recognized that tempo is one of the important characteristics that determine the psychological response to music (Brodsky & Kizner, 2012; Hevner, 1937). Multiple studies have been conducted to study the effects of tempos on various aspects by isolating tempos out of other music elements (Brodsky, 2001; Brodsky & Kizner, 2012; Carpentier & Potter, 2007; Mizoguchi & Tsugawa, 2012). To gain a clear understanding of how tempos affect drivers’ attention and fatigue status, we controlled other music elements by choosing one popular classical music piece with different tempos. This process will limit the generalizability of our research in that our conclusion may not be suitable for all other types of music. However, due to the popularity of both the chosen music and the classical music genre, we believe our results will still be very relevant to a broad variety of music genres.

The arousal hypothesis holds that the arousal effect of music can alleviate fatigue and chronically maintain driving alertness under monotonous road conditions (Brown, 1965). However, the distraction hypothesis suggested acute interfering effect of music stimulation on driver alertness (Turner, Fernandez, & Nelson, 1996). Thus, these two hypotheses each emphasize one side effect of music. Oron-Gilad, Ronen, and Shinar (2008) proposed a dynamic model that integrates these two views and suggests that music has either an arousing or distracting effect on drivers, depending on the dynamic modulation among the properties of the music, the scenario requirements of the drive and the individual differences of drivers. This dynamic model is more inclusive but is still unable to explain the underlying mechanism of how a driver can be both distracted by music and maintain alertness.

Based on the dynamic model, researchers have investigated the effects of different factors of music on driving safety, such as the type of music, volume of music, audio system operation and demographic data of drivers. A simulated road test showed that music could increase drivers’ attention (Venter, 2011). Although previous studies have shown the effects of music on the emotions and attention of drivers, it is unknown to what extent a certain music tempo could change the fatigue and attention of drivers, especially during long-term, real-world, monotonous driving. Music tempo (or sound velocity) is a main factor in the psychoreaction that induces stimulation (Ma, Lai, Yuan, Wu, & Yao, 2012). Using the arousal effect of music to promote drivers’ alertness while avoiding distraction is a complicated but essential research theme. The effect of musical familiarity on drivers has been studied from the perspective of lyrics (Dressel & Atchley, 2008). However, to date, few studies have investigated the effects of music tempo on the physiological status of drivers during real-world, long-term, monotonous driving. Nevertheless, such studies are needed to supplement the research on the dynamic model.

In addition, the arousal hypothesis does not consider the difference between passive fatigue and active fatigue; long-term driving in a monotonous scenario will cause passive fatigue possibly due to the lack of stimulation (Desmond & Hancock, 2001), but long-term driving or sleep deprivation in a complex scenario and changeable scenario will induce active fatigue (Thiffault & Bergeron, 2003a, 2003b). As stated earlier, monotonous driving will subject the driver to a low arousal status, and in this scenario, the lack of driver alertness may lead to severe safety issues (Turner et al., 1996).

To further improve and supplement the arousal hypothesis, Hancock and Warm (2003) proposed the concept of physiological and mental maximal adaptability, built dynamic models of stress and sustained attention and integrated the concepts of arousal and attention into a unified model. In particular, different music tempos affect a driver’s physiological index and driving behaviour differently. In one prior study, Brodsky (2001) experimentally investigated the influence of music tempos on driving using a driving simulator, 20 subjects with a mean age of 32.6 years and defined slow, medium and fast music tempos at 40 to 70, 85 to 110 and > 120 bpm, respectively. Brodsky (2001) found that both simulated driving speed and perceived speed estimates increased consistently with the rise of music tempo. Furthermore, the tempo of background music consistently affected the frequency of virtual traffic violations. Previous physiological research on music tempo has shown that humans who are listening to music have stronger skin conductance responses than those who are not. Faster tempos also trigger stronger physiological excitement (Carpentier & Potter, 2007). Experiments using driving simulation have shown that drivers listening to faster tempo music perceive their driving speed as being below their actual speed and are more prone to collisions, illegal lane crossing and running red lights (Brodsky & Kizner, 2012). Music levels below 80 bpm may encourage safe driving during fast driving situations, such as racing (Mizoguchi & Tsugawa, 2012).

As stated earlier, the dynamic model is more inclusive but is still unable to explain the underlying mechanism of how a driver can be both distracted by music and maintain alertness. Currently, there is still no comprehensive, quantitative study investigating the effects of music tempo on drivers’ fatigue and attention. A frightening proportion of drivers reportedly fall asleep at the wheel while listening to the radio (Oron-Gilad & Shinar, 2000). This self-reporting by drivers cannot be used as a single criterion; instead, more objective data are necessary for a comprehensive assessment. In this study, we used physiological measurements of drivers’ attention while listening to the music of different tempos to examine the effect of music tempo on driving performance and successfully compensated and explored the influencing factors in the dynamic model.

In this study, we hypothesized that different music tempos would either improve or weaken drivers’ fatigue status and attention level. We investigated how different music tempos affect drivers, especially under the challenge of long-distance driving on real-world roads. In the tests, a music piece with four different tempos was played to 16 drivers during up to 140 min of driving on a highway. Drivers’ fatigue status and attention levels were measured. Each driver first drove without any music for 80 min and then was assigned to one of the following four scenarios: no music, slow tempo, medium tempo or fast tempo. The mental load and eyeball movement of each driver, which served as indicators of fatigue and attention level, respectively, were recorded using an electroencephalogram (EEG) and an eye tracker, respectively.

We found that the effects on the fatigue status and attention level of drivers differed among the four music tempos. When a driver was tired, playing any of the music would improve his or her mental status for the first 30 min; however, after 30 min, the results greatly differ. We also explored the underlying mechanism of how music tempo affects alertness maintenance in the dynamic model. The avoidance of driving fatigue and maintenance of attention are essential for driving safety. Our findings will offer empirical evidence that can help drivers to select appropriate music and improve their long-distance driving safety.

## Methods

This study focused on the relationships between two dependent variables (fatigue and attention) and an independent variable (music tempo). We carefully chose three variations of one music track that was remixed at different tempos as the independent variable. Sixteen people were recruited to listen to these various tempos during a prolonged driving experiment. The drivers’ brain and body indices were recorded to measure their fatigue and attention level.

### Participants

The 16 participants (8 males and 8 females) all had a current driving license and self-reported over 10,000 km of driving experience (range: 1–10 years, mean 3.8 years, standard deviation [*SD*]: 3.0 years). They were 20 to 35 (*M* = 26, *SD* = 3.2) years old. All drivers had visual acuity of 1.0 or above. They were all in good health conditions and had no hearing impairments. None of them had received professional music training. Before the experiments, the participants completed a questionnaire on their gender, age, driving experience, visual acuity, physical status, and mental health.

### Materials

**Figure 1.**
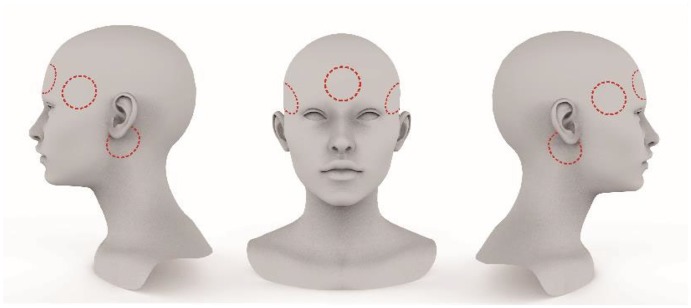
The positions of the EEG electrodes.

We recorded the drivers’ physiological status and eye movement data using a biofeedback instrument and an eye tracker. A portable 10- to 16-channel Spirit-10markII biofeedback instrument (Precision: 24 bits; sampling rate up to 8,192 sample/s) was used to record the drivers’ physiological status, including an electrocardiogram, an EEG and electromyography. According to product manuals and international standards (Dressel & Atchley, 2008), we placed five electrodes on each driver, with one each at the left, middle and right spots of the anterior forehead and two at the spots behind the bottom of each ear (the red circles shown in Figure 1). The EEG feedback and EEG activities (amplitude and frequency) at different body positions were measured to provide feedback signals (α wave, β wave, θ wave and sensorimotor rhythm [SMR] wave). The SMR was used to measure variation in the driver’s attention (Shafaei, Hajkazemi, Desnoyers, & Aghayev, 2016). In addition, a Careland® event data recorder (video resolution 1080p; photo pixel: 12 million) was installed on the rearview mirror to record the driving speed, traffic video and driving trajectory.

**Figure 2.**
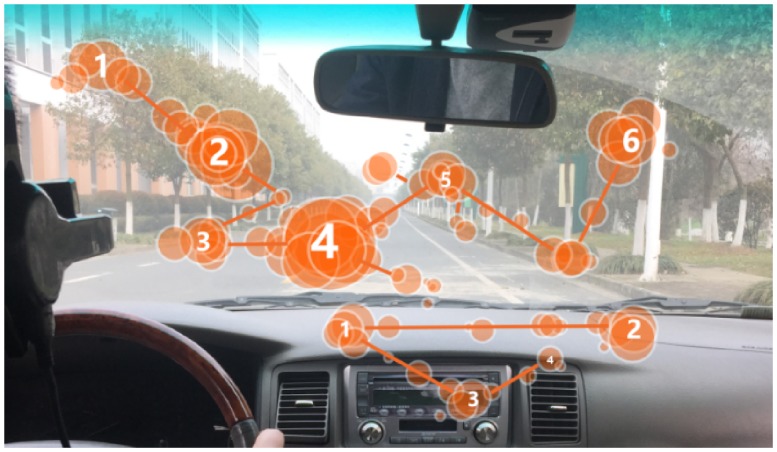
Driver glance orbits and the scene of driving experiments.

We used an EyeGuide® mobile tracker to record real-time eye gaze movement. First, all eye movement data were recorded in real time by the real-time data capturing application of EyeGuide®. Then, after the experiment, we used the software EyeGuide® Analyze to analyse the data. In EyeGuide® Analyze, we set the eye gaze stay time at 100 ms. Only if the eye focused on a point for at least 100 ms did we consider the point as an eye gaze focus. The wearing of the eye tracker did not interfere with the driver while listening to music. Figure 2 shows a scan path example of the eye gaze focus of one driver. Saccade can be measured from the distances among eye gaze focus points. Saccade ratings were analysed using EyeGuide® Analyze. More specific instructions for users can be found at https://eye.guide/. Finally, we used mixed analysis of variance (ANOVA) to analyse the relations of different tempos with all recorded data, including *R* values, saccade speed, SMR and visual search range.

To prevent drivers from being confused by the cognition psychology of being unfamiliar with the music that they are listening to, thereby interfering with the drivers’ visual behaviours (Salamé & Baddeley, 1989), we selected one of the most popular Chinese classical music pieces, “Moonlight Over the Spring River.” This music originated approximately three hundred years ago and has been developed in many different variations. According to a music repository (music.baidu.com), there are up to 322 different remixes of “Moonlight Over the Spring River.” People have heard this musical piece in various locations, including elementary music class or as background music in restaurants. Musicians have created their own interpretations of this music for different contexts using various instruments. There is no default version for this music. People would not be surprised when listening to a different version of this music. Therefore, this music was selected to ensure that the independent variable was very clear and to largely eliminate cofounding variables related to music, such as melody and genre. We selected three versions, which corresponded to our slow-tempo (40–70 bpm), medium-tempo (85–110 bpm) and fast-tempo (> 120 bpm) categories. Music information, including tempo, artist and key, was analysed through the music software MixMeiser Studio (Table 1), which reported the tempos of these remixes were 42, 92 and 122 bpm, respectively. In addition, in the tests, we did not shift from one tempo to another but rather from no music to one selected tempo. Therefore, the effect of tempo change was minimal. All three remixes were approximately 5 min in length (5:11, 5:05 and 5:20 for the slow-, medium- and fast-tempo remixes, respectively). In China, many stores and restaurants use this music as background music and play it in loops. During the driving experiment, the music was repeatedly played at a volume through the car player, which was selected because setting the intravehicle music loudness at a medium volume < 80 dB is most favourable for safe driving (Mizoguchi & Tsugawa, 2012).

**Table 1.** Music Information (Title, Artist, Besat Per Minute (BPM)) Through MixMeiser Studio.

No.	Title	Artist	BPM	Transition
1	Moonlight Over the Spring River	Central National Orchestra	42.6 (100%)	Beatmix8
2	Moonlight Over the Spring River	Leishi Chen	91.7(100%)	Beatmix8
3	Moonlight Over the Spring River	Kuizhi Yu and Shengsu Li	122.1(100%)	Beatmix8

### Experimental Conditions

All 16 drivers completed one full set of experiments, that is, each driver drove four times and listened to the no-tempo, slow-tempo, medium-tempo and fast-tempo music. To eliminate the impact of the music sequence, we used a Latin square sampling method to differently order the music tempos for each driver, aiming to examine how music affects fatigue during prolonged driving. Because the safe limit for monotonous highway driving is 80 min (Li, Su, & Lu, 2017), each driver was asked to drive for 80 min without music, thereby reaching a certain level of tiredness; then, they listened to the music of a certain tempo (or continued in silence for the no-music condition) for 60 min. The driver’s physiological indices were continuously recorded. Sixty-four driving experiments (4 drives for each of 16 participants) were conducted over 64 days, and drives were performed 2 times per day from 7:00 to 11:00 a.m.

All experiments were conducted via the same driving route on a monotonous and low-complexity road. The conditions were as follows: traffic flow below 50 vehicle/hr/lane; peak hour factor = 0.95; smooth safe road; no crossings or traffic lights; a monotonous landscape; driving for 140 min; average velocity at 100 to 120 km/hr (120 km/hr is the velocity limit for small vehicles on highways in China). The experiments were conducted during the fall in Jiangsu, China. The weather was mostly clear, with comfortable temperatures (mostly between 18°C and 25°C).

### Procedures

The participants were given the same instructions to keep the study conditions as consistent as possible. All participants were asked to (a) have sufficient sleep and not consume energy drinks or alcohol before the driving tests and (b) wear comfortable clothing suitable for driving under ambient temperatures (most people wore t-shirts, jeans and walking shoes).

The same procedures were followed for all driving experiments:

Procedure 1: The participants received short-term training to learn the objective, process and tasks of the tests. This training was conducted to ease their psychological stress. The vehicle seat and wheel positions were adjusted to ensure that the drivers were in their most comfortable driving positions. All experimental equipment, including the EEG, car music player and eye tracker, was adjusted accordingly.
Procedure 2: Each driver first drove for 80 min without music (Li et al., 2017) and then drove for 60 min while listening to a selected music tempo at a volume of 65 dB (or in silence for the no-music condition). The driver’s body data started recording at the 75-min mark and then were tracked every 5 min until the end of the experiment. These data were used to examine the driver’s status while listening to music with different tempos.

## Data Analyses and Results

The temporal changes of physiological indices under different music conditions (no music, slow tempo, medium tempo and fast tempo) were compared. Therefore, data were analysed via two-way mixed ANOVA. Specifically, an overall mixed ANOVA was run to verify the significance of the multivariate test. The effect sizes were reported as partial eta squared, and a 0.5 level was set for statistical significance.

### Effect of Music Tempo on Fatigue

The EEG spectrum *R* value and average eye movement speed can be used as indicators of fatigue (Yuan, Lai, Wu, & Yao, 2009). We examined the relationship of the *R* value and saccade speed with the four different music conditions under two driving periods (80–110 min and 110–140 min) through mixed ANOVA.

#### *R* value

Driver fatigue leads to a reduction in β waves and an increase in α waves. The slow θ wave dominates when the fatigue state transitions to the sleepy state (Akerstedt, Kecklund, & Knutsson, 1991). *R* = (α + θ)/β on the EEG spectrum (Shao-Bin, Li, & Wang, 2009), which is an α valid indicator of fatigue during a monotonous driving task (Jap, Lal, Fischer, & Bekiaris, 2009), was used to measure driver fatigue status. During the experiments, drivers’ wave levels were continuously recorded. For a comprehensive analysis and comparison, the mean and SD of the *R* values were computed every 5 min.

**Figure 3.**
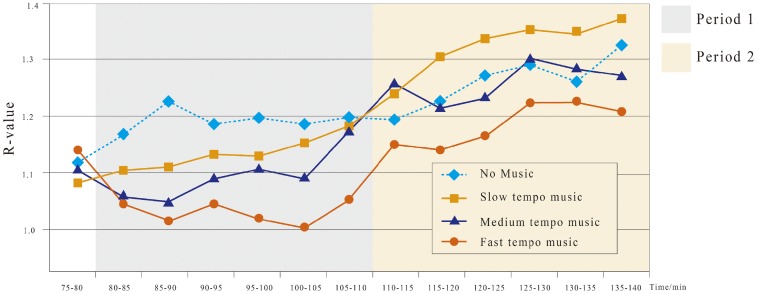
*R* value curves for the four tempos during 75 to 140 min.

A clear distinction appears at approximately 110 min, when the curves intersect. We define Period 1 as 80 to 110 min and Period 2 as 110 to 140 min. The mixed ANOVA shows that the *R* values are significantly different at Period 1 (Figure 3). During Period 1, the *R* values at the slow tempo (*M* = 1.14, *SD* = 0.03), medium tempo (*M* = 1.09, *SD* = 0.04) and fast tempo (*M* = 1.05, *SD* = 0.03) are all significantly smaller than those of no music (*M* = 1.18, *SD* = 0.02). During Period 2, the *R* values increase and continue to differ significantly (Figure 3). The *R* values of the slow tempo (*M* = 1.33, *SD* = 0.05) and fast tempo (*M* = 1.19, *SD* = 0.03; both *p* < .05), but not the medium tempo (*p* > .05), are significantly different from those of no music (*M* = 1.28, *SD* = 0.04).

#### Mean saccade speed

The average saccadic speed (degree/s) is defined as the saccadic distance (angle) within a second. Saccade speed reflects a driver’s ability to address new information and respond to new objects on the road. The average saccadic speed during the fatigue state is significantly lower than that of the nonfatigue state. The mean and SD of the average saccadic speeds were also computed every 5 min.

**Figure 4.**
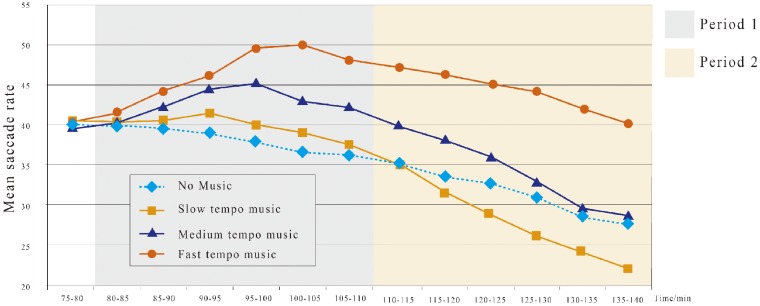
Mean saccade speed change curves for the four tempos during 75 to 140 min.

We also identify two distinct saccadic periods. The curves intersect from 110 to 115 min. Mixed ANOVA shows that the average saccadic speeds for the slow tempo (*M* = 39.75, *SD* = 1.33), medium tempo (*M* = 42.93, *SD* = 1.75) and fast tempo (*M* = 46.53, *SD* = 3.24) are all significantly larger than those of no music (*M* = 38.12, *SD* = 1.60) during Period 1 (all *p* < .05; Figure 4). Within Period 2 (Figure 4), the music tempo significantly affects the average saccadic speed. The average saccadic speed for the slow tempo (*M* = 27.87, *SD* = 4.86) is significantly smaller, while those for the medium tempo (*M* = 34.23, *SD* = 4.62) and fast tempo (*M* = 44.02, *SD* = 2.62) are significantly larger than that of no music (*M* = 31.27, *SD* = 2.90; all *p* < .05).

#### Comparison of music with no music

As shown in Figures 3 and 4, some curves intersect at approximately 110 min. This result prompted us to divide the entire period into two periods (80–110 and 110–140 min). Mixed ANOVA shows that these values change significantly across the two driving periods and four music conditions (Table 2). We then further compared the three music tempos with the no-music condition. For both periods, the mean saccade speed varies significantly (*p* < .05). During Period 1, the saccade speed of all three music conditions was significantly higher than that of no music (*p* < .05).

**Table 2.** *R* Value and Mean Saccade Speed (Mean and SD) for the Four Tempos During 80 to 140 Min.

	*R* value				Mean saccade speed			
	80–110 min		110–140 min		80–110 min		110–140 min	
Music	*M*	*SD*	*M*	*SD*	*M*	*SD*	*M*	*SD*
No music	1.18	0.02	1.28	0.04	38.12	1.60	31.27	2.90
Slow music	1.14	0.03	1.33	0.05	39.75	1.33	27.87	4.86
Medium music	1.09	0.04	1.25	0.03	42.93	1.75	34.23	4.62
Fast music	1.05	0.03	1.19	0.03	46.53	3.24	44.02	2.62

### Effects of Music Tempo on Quality of Attention

The SMR and visual search rate were recorded to examine the quality of attention.

#### SMR

Attention is very susceptible to both the executive task at hand and the environment. A boring situation such as driving on a monotonous highway is likely to lower attention, while music could enrich the driving experience and increase attention. The SMR identified by M.B. It refers to the brain waves in the sensory cortex and motor cortex of the brain. SMR brain wave amplitude is an effective method for measuring a driver’s attention (Shafaei et al., 2016). Vernon et al. (2003) studied the effects of neurofeedback frequency on cognitive performance and found that the SMR of learning self-control can maintain the readiness states of perception and attention. Further research showed that SMR was related to motion inhibition, attention focusing, working memory and other mental load factors (Gruzelier, Egner, & Vernon, 2006). A person will produce a stronger SMR amplitude when the corresponding sensorimotor areas are idle (e.g., during an immobile state). If the sensory or motor areas are activated, the SMR amplitude will decrease. As reported, even weak persistent environmental irritation can improve the arousal level of the human brain network, thereby helping humans maintain stable attention. In this study, a biofeedback instrument was used to record the EEG SMR amplitude.

**Figure 5.**
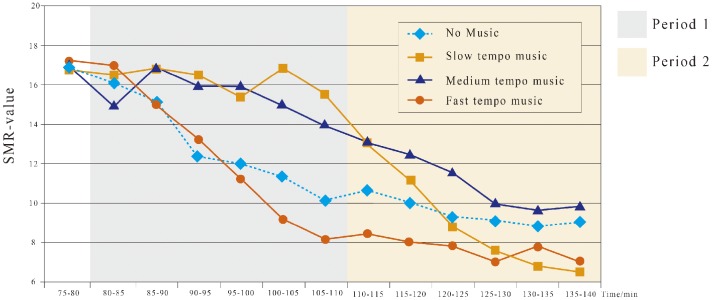
Amplitude change of SMR under four tempos during 75 to 140 min. SMR = sensorimotor rhythm.

As shown in Table 3, the SMR wave amplitudes under slow-tempo, medium-tempo and fast-tempo music are significantly different from those of no music (all *p* < .05). Mixed ANOVA shows that the SMR amplitudes under the slow tempo (*M* = 16.25, *SD* = 0.63) and medium tempo (*M* = 15.50, *SD* = 1.05), but not the fast tempo (*M* = 12.32, *SD* = 3.39), are both significantly higher than that of no music (*M* = 12.88, *SD* = 2.29) within Period 1 (Figure 5). Within Period 2, the SMR amplitude under no music further declines stably (Figure 5). Moreover, the SMR amplitude for the medium tempo (*M* = 11.05, *SD* = 1.46) is significantly larger, and the SMR amplitudes for the slow tempo (*M* = 8.82, *SD* = 2.60) and fast tempo (*M* = 8.00, *SD* = 0.56) are significantly smaller than that of no music (*M* = 9.47, *SD* = 0.69; all *p* < .05).

**Table 3.** SMR and Visual Search Values (Mean and SD) for the Four Tempos During 80 to 140 Min.

	SMR				Horizontal visual search				Vertical visual search			
Music	80–110 min		110–140 min		80–110 min		110–140 min		80–110 min		110–140 min	
	*M*	*SD*	*M*	*SD*	*M*	*SD*	*M*	*SD*	*M*	*SD*	*M*	*SD*
No music	12.88	2.29	9.47	0.69	18.8	2.12	29.18	3.50	29.18	3.50	18.8	2.12
Slow music	16.25	0.63	8.82	2.60	16.89	1.62	31.45	5.67	30.65	3.27	16.58	3.95
Medium music	15.50	1.05	11.05	1.46	16.5	0.49	22.18	3.60	31.73	1.09	24.42	2.72
Fast music	12.32	3.39	8.00	0.56	26.33	2.33	34.25	2.45	24.25	2.45	16.36	2.28

*Note*. SMR = sensorimotor rhythm.

#### Visual search field

Visual search is a major method of human information acquisition by gazing and glancing at the stimuli (Tsimhoni, Smith, & Green, 2004; Yantao, Yuchang, & Xue, 2006). The horizontal and vertical visual search ranges characterize the extents to which a driver can notice the targets in their field of view. Victor, Harbluk, and Engström (2005) used the SD of composite line of sight (namely, the composite value of SDs of horizontal and vertical lines-of-sight calculated from the Pythagorean theorem) as a comprehensive evaluation index of the visual search scope of drivers. As reported, the driver’s line of sight is focused on a certain portion of the road (percent road centre). Given the driver’s operation time, percent road centre significantly declines throughout each drive (Victor et al., 2005). We can easily find the eye gaze on the four directions of top, bottom, left and right. The horizontal search range is the eye viewing angle from the left end to the right end. The vertical search range is the viewing angle from the top point to the bottom point. The total field view is simplified as a rectangular area defined by the four limits of the left/right/top/bottom.

**Figure 6.**
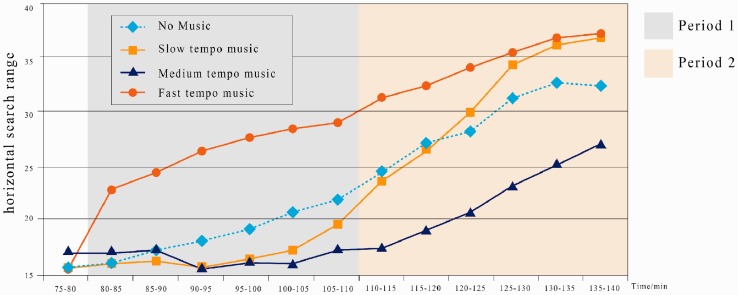
Change of horizontal search range under four tempo conditions during 75 to 140 min.

Mixed ANOVA shows that the horizontal search ranges for the slow tempo (*M* = 16.89, *SD* = 1.62) and medium tempo (*M* = 16.5, *SD* = 0.49), but not the fast tempo (*M* = 26.33, *SD* = 2.33), are both significantly smaller than that of no music (*M* = 18.8, *SD* = 2.12) within Period 1 (both *p* < .05; Figure 6). Within Period 2, the horizontal search range under no music rose consistently (Figure 6). In conclusion, music tempo significantly affects the horizontal search range. Mixed ANOVA shows that the horizontal search range for the medium tempo (*M* = 22.18, *SD* = 3.60) is significantly smaller and those for the slow tempo (*M* = 31.45, *SD* = 5.67) and fast tempo (*M* = 34.25, *SD* = 2.45) are significantly larger than that of no music (*M* = 29.18, *SD* = 3.50).

**Figure 7.**
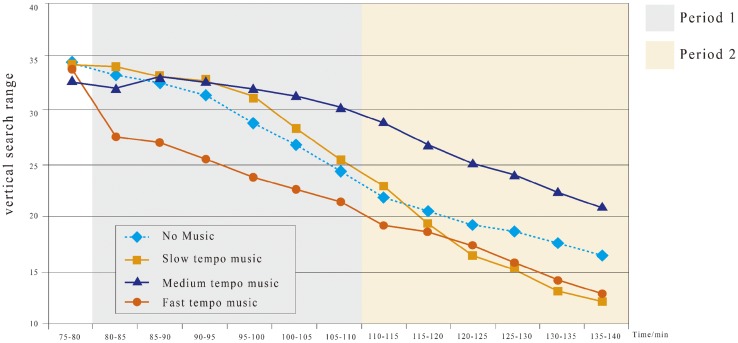
Change of vertical search range under the four tempo conditions during 75 to 140 min.

Mixed ANOVA shows that the vertical search ranges for the slow tempo (*M* = 30.65, *SD* = 3.27) and medium tempo (*M* = 31.73, *SD* = 1.09), but not the fast tempo (*M* = 24.25, *SD* = 2.45), are both significantly higher than that of no music (*M* = 29.18, *SD* = 3.50) during Period 1 (Figure 7). During Period 2, the vertical search range for no music consistently declines (Figure 7). Moreover, the music tempo significantly affects the horizontal search range. The vertical search range for the medium tempo (*M* = 24.42, *SD* = 2.72) is significantly larger, but those for the slow tempo (*M* = 16.58, *SD* = 3.95) and fast tempo (*M* = 16.36, *SD* = 2.28) are both significantly smaller than that of no music (*M* = 18.8, *SD* = 3.50).

#### Comparison of music with no music

The two periods (80–110 min and 110–140 min) were further analysed to examine different music conditions. Mixed ANOVA shows that these values change significantly across the two driving periods and four music conditions (Table 3). The medium-tempo music significantly outperforms the other music conditions (slow, fast and no music), with a confidence level of *p* < .05.

## Discussion

The earlier data analysis suggests that medium-tempo music may be the most effective tempo to maintain drivers’ attention because the value changes of SMR and the visual search field under the medium tempo are the smallest among all four conditions. Music can improve humans’ attention, memory and learning abilities (Barreto, Taele, & Hammond, 2016), but humans have limited cognitive capacities. Significant cognitive workloads, including driving, consume these limited attention resources. A proper vigilance-maintaining task should be able to improve drivers’ arousal without causing distraction. Our study suggests that medium-tempo music most effectively succeeds in doing so. Compared with all other music conditions, the medium-tempo music maintains drivers’ attention and fatigue levels for the longest period of driving. Slow-tempo music is generally expressed as a feeling of calmness and is often used in therapies composed of relaxing or sleeping. The level of fatigue is typically associated with the level of sleepiness. Therefore, slow music will increase the level of fatigue and sleepiness. High-tempo music is often considered energetic (Venter, 2011) and can keep people alert, which will reduce fatigue. However, high-tempo music may stimulate drivers too much. When a driver is stimulated over a longer period of time, she or he may get tried from continued overexcitement, which could cause a further reduction in attention level. For slow-tempo music, a driver will have a higher attention level during a short beginning period. However, in the long run, because it can calm people down, the driver will feel sleepy/fatigue, which could cause their attention level to drop sharply.

### Effects of Tempo on Fatigue

Fast-tempo music might be best at reducing driver fatigue because its *R* value and saccade rate outperform the other tempo conditions. As shown in Table 4, the *R* values for fast-tempo music are within the range of 1.00 to 1.25 during both periods, which correspond to the awakened and slightly fatigued states, respectively (Yuan et al., 2009). Within Period 1 (80–110 min), moving from the no-music condition to the fast-tempo condition, the driver’s fatigue condition was significantly relieved, as shown by the noticeable decrease in *R* values. This result aligns with previous research showing that a change in music tempo led to a more severe change in α-wave values. Medium-tempo music also improved fatigue states during Period 1. However, for a longer time in Period 2, the fatigue condition declined consistently. The slow-tempo music did not enhance the fatigue during Period 1. During Period 2, fatigue further deteriorated to the lowest state. In conclusion, fast-tempo music stimulates drivers more than slow- or medium-tempo music, which is consistent with previous studies on fast-tempo music and physiological excitement.

**Table 4.** Fatigue Evaluation Based on EEG (Yuan et al., 2009).

Driving fatigue	*R* value
Conscious	*R* < 1.15
Low fatigue	1.15 ≤ *R* < 1.25
Middle fatigue	1.25 ≤ *R* < 1.35
High fatigue	1.35 ≤ *R*<1.45
Sleep	*R* ≥ 1.45

*Note*. EEG = electroencephalogram.

Human eyes make saccadic movements while scanning the surrounding environment. Our results show that the mean saccade speed under the no-music condition gradually declines with time, indicating that the driver’s fatigue is worsened during prolonged driving (Stasi et al., 2012). For drivers listening to music, the mean saccade speeds increased during Period 1 but gradually dropped during Period 2. After a short period of driving, saccade data under music scenarios are generally higher than that under the no-music scenario (He, Becic, Lee, & Mccarley, 2011). For prolonged driving, the drivers’ saccade speed drops significantly under slow-tempo music, which is correlated with high-level fatigue (Di Stasi et al., 2015). We can see that both high-tempo and medium-tempo music can keep drivers alert. Their saccade speeds remain higher than those in the no-music scenario. As high-tempo music stimulates and excites the drivers more, the saccade speed is the highest among all the conditions. Under normal circumstances, the time needed to maintain the mean saccade speed is 0.2 to 0.3 s. If a driver’s eyes were closed for 0.5 s, he or she would be very likely to engage in a traffic accident. Our experiments show from a qualitative perspective that faster tempo music tends to help drivers avoid fatigue.

Moreover, the average saccadic speed significantly dropped with prolonged operation time and that of fast-tempo music was consistently the highest among all music tempos. This observation is consistent with those of Cazzoli et al. (2014). This finding is also consistent with the *R* values, which further proves the efficiency of fast-tempo music on physiological arousal.

### Effects of Tempo on Attention

**Figure 8.**
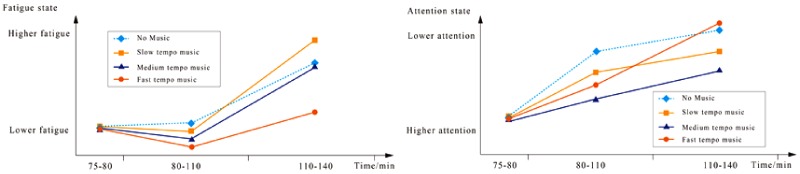
Fatigue and attention state change curves for the two driving periods.

Human brains react significantly to music, as represented by brain waves (Kaliakatsos-Papakostas, Andreas, & Vrahatis, 2016). Among brain waves, the θ wave typically indicates emotional suppression or the resting state; the α wave represents an awake and relaxed state; the β wave reflects mental stress or emotional excitement; the SMR wave or low β wave reveals the attentive state. As shown in Table 5, during a prolonged driving task, the driver’s quality of attention decreases. Fast-tempo music will further deteriorate the quality of attention (Cheng et al., 2015). Fast-tempo and high-intensity music (e.g., rock and roll) directly affect driving safety and operational efficiency (Venter, 2011). Slow-tempo and low- to medium-intensity background music contribute to increasing the nervous state, which improves the quality of attention and driving performance. Based on the changes in the *R* values in Table 4 and the changes in the SMR in Table 5, as well as the fatigue and attention evaluation based on EEG, we obtained Figure 8. The medium-tempo music improves attention, while slow-tempo music is effective only during the first period (Figure 8). Therefore, medium-tempo music is the best for activities that require heightened attention and concentration. This conclusion aligns with the results of Dalton and Behm (2007).

**Table 5.** Table for Attention Level Evaluation Based on EEG (Cheng et al., 2015).

Driving attention	SMR/μV
Inattention	Amp < 10
Low attention	10≤ Amp <15
Middle attention	15≤ Amp <20
High attention	Amp ≥ 20

*Note*. EEG = electroencephalogram; SMR = sensorimotor rhythm.

Tempo significantly affects the changes in horizontal and vertical search ranges. Our experiments show that the fastest tempo music enlarged the visual search range of drivers; however, after a long driving time, the results did not differ much from those of the slowest tempo. In contrast, the medium tempo led to the smallest change in the visual searching range. This smaller change may indicate that the driver’s attention level also changes within a smaller range. Zhang, Kaber, Rogers, Liang, and Gangakhedkar (2014) found that as the time of fixating at the road centre by the driver was prolonged, the time of fixating at the two sides of the road and the intravehicle facilities was shortened accordingly, or namely, the number of glances decreased with the intensified cognition distraction. This outcome may explain the association of visual search fields with attention level. According to Figures 6 and 7, we can see that under different music tempos, the changing amplitude of the medium tempo within 80 to 140 min was the most stable and the smallest compared with the other music tempos. The other tempos all caused much more significant changes. Generally, a driver’s early attention level should be better than that at a later time. The smaller the change over time, the better the driver maintained his or her attention. Similarly, when the drivers were under a high-cognition load, the distribution of horizontal fixation points was significantly reduced, while that of vertical fixation points increased moderately, indicating the drivers, through enlarging the visible distance, adapted to the cognitive overload (Richard, 2012). It was indicated that, as the attention of the drivers declined, the glance extent was increasingly narrowed, while the medium-tempo music maintained a longer temporal effect on enhancing the drivers’ attention and glance extent.

**Figure 9.**
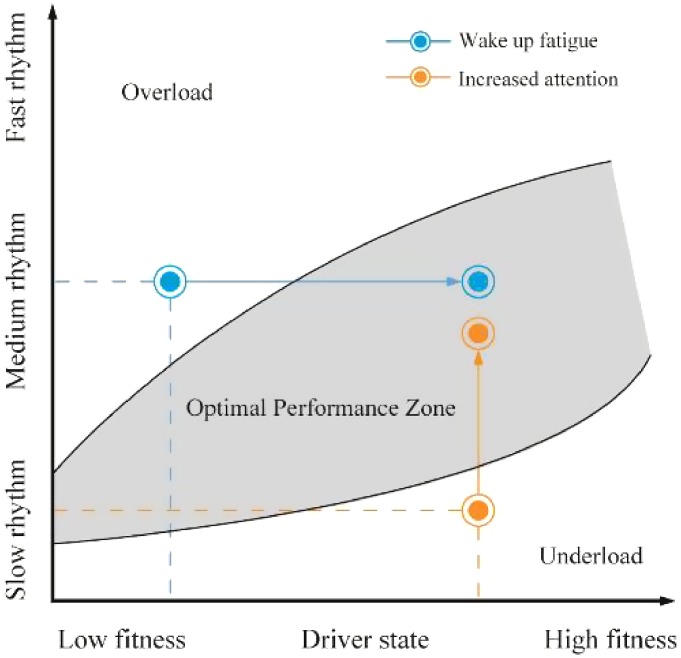
The interventions of the drivers under the conditions of overload and underload.

In this study, based on the dynamic model from Oron-Gilad et al. (2008), we proposed that the mental workload of the drivers was a function of driver fitness and the driving scenario requirements. The workload of the drivers was generally divided into three levels: low load, optimal load and excessive load. As shown in Figure 9, the *y* axis is the variation of the music tempo, and the *x* axis is the driver fitness, such as driving experience, personality, emotion, motive and pressure. When the required music tempo is appropriate and the driver fitness is in good condition, the driver could drive the best. A driver’s workload consumes their attention and thereby intensifies physiological arousal; however, load deficiency will lead to a low arousal status and thus reduce alertness. Appropriately increasing the alertness-maintaining tasks may improve the arousal and alertness performances, but alertness-intervention tasks should be established according to the workload level of drivers. For example, if a driver is fully concentrated on extra alertness-maintaining tasks, he or she will feel the excessive load, which leads to distraction and a lack of alertness. Thus, extra alertness-maintaining tasks should be established to improve driver arousal without causing distraction. This requirement is seemingly fully met by listening to music when controlled at a suitable tempo (Oron-Gilad et al., 2008). Through our tests on music tempo, we explored the concrete situational driving demands of the dynamic model and successfully verified the effects of music tempo on the workload of drivers.

### Generalizability

The findings of this study could have limited generalizability to music pieces in other genres. First, from the perspective of existing music pieces, more than 8,000 music pieces stored in the music repository (music.baidu.com) belong to the Chinese classic music genre, such as “Moonlight Over the Spring River.” These music pieces are extremely popular; more than 6,000 music pieces are downloaded with a high frequency (download > 140,000 times). Second, music pieces may be different regarding their instrument or style, but there are several key fundamental features that exist in all music pieces. Tempo, one of the basic constructs that define the speed of music playing, can provide a clue to the musical feel the composer intended and is critical to the mood of a musical piece. Hevner (1937) noted that among six music elements (mode, melody, rhythms, modern dissonant harmonies vs. classical consonance, pitch, tempo), tempo plays the highest importance in carrying music expressiveness. Many genres (especially electronic and dance music) are defined by the signature tempo. Therefore, although we used only one musical piece, due to the universal characteristics of tempo in musical pieces, the conclusions about tempo can be generalized to many other types of music pieces. In addition, tempo has been studied extensively in various environments, such as shopping centres and restaurants. In general, a faster tempo makes people excited or behave faster, which aligns well with our findings.

From the perspective of new music creation, according to the dynamic model and together with the thresholds of enhancing attention and relieving fatigue, driving safety and performance can be enhanced by controlling music tempos. Brodsky and Kizner (2012) developed a set of intravehicle background music that adjusts driving alertness. Their hypotheses state that if such music is limited to intravehicle listening, it would not induce thinking or recalling from drivers, because such music has no lyrics and avoids language processing, that is, the melody is obscure and cannot be followed. The music has excellent musical quality and variable chords accompanied by complex and delicate syncopation but lacks specific and clear tempos. In this study, the tempo creation was optimized, which could be popularized to more music pieces.

### Limitations

We acknowledge that our findings regarding tempo represent only a general trend rather than a specific function. For music in general, new musical forms are consistently being created by composers. The audience is becoming increasingly sophisticated. Because music is a complex combination of many interacting elements, tempo cannot represent the accurate effect of a composition as a whole. Although a composition is ideal for studying syntheses and the combinations of music elements, its infinite variety of moods and feelings makes it impossible to trace all these variations. Thus, in this study, we explored how music tempo alone affects driving fatigue or attention levels.

This study has several limitations. First, the drivers were all in their 20s or 30s. Compared with these younger drivers, senior drivers may be more patient and calmer; thus, they may be impacted by music differently. Second, we played only 1 hr of music after 80 min of no-music driving. This limitation was primarily due to the 1-hr battery duration limitation of our equipment. Once the battery capacity improves, it would be worthwhile to track prolonged driving experience from the beginning to the end of each drive. Moreover, in terms of music piece selection and to prevent the interfering factors, we selected only one music piece; also, by changing the velocity of the music tempo, we made the drivers remember and psychologically depend on the familiarized music, which would affect the drivers’ behaviours and thereby weaken the impacts due to the music tempo velocity. One reason we choose this musical piece was to eliminate confounding variables. There are many different styles/genres of music. Different people may simply prefer one genre but dislike another genre. Previous work has compared different music genres, for example, jazz or classical, regarding their effect on driving. This selected piece in this study is a beautiful, well-known, popular piece in China. Thus, we do not need to address the user’s preference in this study. Another reason we selected this piece is that it can be played in loops without making the audience feel tired or bored. In many places (e.g., stores), this music has been played in loops as background music. However, such isolation of tempo from other music elements will limit the generalizability of this research to other music genres.

There is still much to do regarding studying how other music elements and their interactions affect driving. We will expand on the findings of music tempo and conduct comprehensive studies by adding other music elements that affect driving safety, such as tone frequencies, instrumental ranges, arrangements, voicing textures, rhythm and intensity. In the future, we may consider the following experiment setting: a different combination of music features, weather conditions, higher speeds on the highway, a broader range of driver ages and longer driving durations.

## Conclusions

Driver fatigue and attention levels under different music tempos (slow tempo, medium tempo, fast tempo and no music) were studied during a 140-min real-life driving experiment on a monotonous highway. Drivers’ EEG data, eye saccade speed, SMR and visual searching range were recorded to measure their fatigue and attention states. After 80 min of no-music driving, the drivers listened to 60 min of music at different tempos. We observed the following: (a) Medium-tempo music helps to improve fatigue and attention levels in the long run; (b) slow-tempo music improves only the quality of attention for a short time and significantly deteriorates the fatigue status and attention level compared with the no-music condition; and (c) fast-tempo music helps to relieve long-term fatigue but further deteriorates the drivers’ attention compared with the no-music condition.

This study provided a useful reference for drivers who listen to music and for music stations that provide music for drivers. When a driver fails to stay alert and suffers from passive fatigue on a monotonous road, the tempo could be an essential indicator for choosing music so that he or she can improve his or her attention and overall driving safety. As a direct conclusion for this work, when listening to classical music while driving, medium-tempo music might be the best choice.
